# Structural architecture of a hybrid β-barrel assembly machinery BAM-TAM complex in *Borrelia burgdorferi*

**DOI:** 10.1016/j.jbc.2026.113267

**Published:** 2026-06-19

**Authors:** Kalvis Brangulis

**Affiliations:** Tick-Borne Pathogens Research Group, Latvian Biomedical Research and Study Centre, Riga, Latvia

**Keywords:** BAM complex, TamB, β-barrel assembly machinery, outer membrane protein biogenesis, Borrelia burgdorferi

## Abstract

The β-barrel assembly machinery (BAM) inserts outer membrane proteins (OMPs) into the outer membrane of Gram-negative bacteria. In well-studied organisms such as *Escherichia coli*, BAM consists of BamA and four accessory lipoproteins (BamB-E) that coordinate substrate recognition, membrane remodeling, and β-barrel insertion. In the Lyme disease spirochete *Borrelia burgdorferi*, however, the BAM system is simplified, comprising only BamA (BB0795), BamD (BB0324), and BamB (BB0028), while lacking BamC and BamE. In addition, *B. burgdorferi* encodes a TamB homolog (BB0794), suggesting a hybrid BAM-TAM organization. Here, we define the structural architecture of the spirochetal BAM-TAM system. AlphaFold3 modeling predicts that BamD binds to BamA periplasmic domains POTRA4-5, while BamB interacts with POTRA1, POTRA3, POTRA4, and POTRA5, forming a closed periplasmic ring distinct from the enterobacterial arrangement. Microscale thermophoresis confirms micromolar-affinity binding of BamD to BamA POTRA3-5, no detectable interaction with POTRA1-3, and loss of POTRA3-5 interaction upon mutation of key interface residues. Crystal structures of BamA POTRA1-2 and POTRA2-3 further define the architecture and flexibility of the periplasmic region. Strikingly, structural modeling of the full complex suggests that the C-terminal β-strands of TamB complete the BamA β-barrel through β-strand augmentation, forming a continuous hybrid barrel with a hydrophobic exterior and hydrophilic interior. This arrangement may provide a direct structural link between periplasmic protein handling and membrane insertion. Together, these findings support a reorganized BAM architecture in *B. burgdorferi* and suggest that the hybrid BAM-TAM organization is conserved across Lyme disease-associated *Borrelia* species, providing a framework for understanding outer membrane assembly in spirochetes.

Diderm bacteria rely on highly conserved molecular systems to assemble outer membrane proteins (OMPs), yet spirochetes, including *Borrelia burgdorferi*, present distinctive adaptations in outer membrane organization. The outer membrane (OM) of *B. burgdorferi*, the etiologic agent of Lyme disease, contains numerous surface-exposed lipoproteins but remarkably few integral β-barrel OMPs, approximately tenfold fewer than *Escherichia coli* (1, 2). Despite their low abundance, proper biogenesis and insertion of these β-barrel OMPs are essential for viability, membrane integrity, and host adaptation (3, 4). In diderm bacteria, β-barrel OMP assembly is mediated by the β-barrel assembly machinery (BAM), a conserved multiprotein complex centered on BamA (5, 6). BamA, a member of the Omp85 superfamily, constitutes the membrane-integrated core of the complex (6). Structural studies have revealed that *E. coli* BamA contains a 16-stranded transmembrane β-barrel and five N-terminal periplasmic polypeptide transport-associated (POTRA) domains (7) (Fig. 1*A*). In the fully assembled *E. coli* BAM complex, the POTRA domains together with BamD form a ring-like periplasmic architecture positioned beneath the β-barrel, creating a coordinated scaffold for substrate engagement and accessory protein docking (8) (Fig. 1*A*).

**Figure 1:**
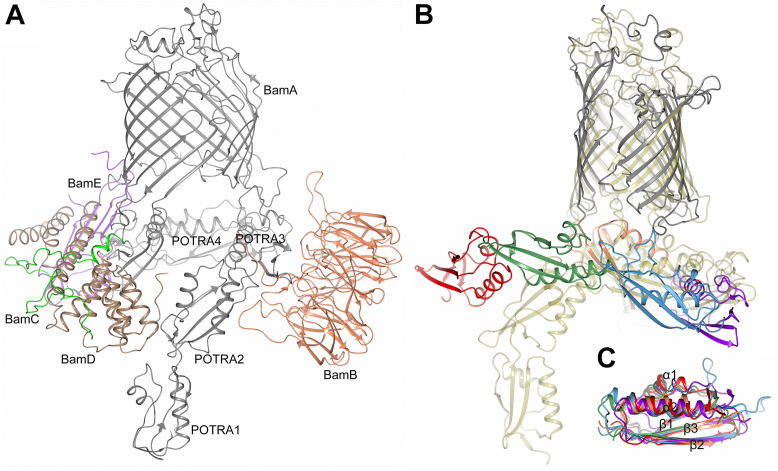
**Structural organization of BamA and the BAM complex**. *A*, Structure of the *E. coli* BAM complex (PDB ID: 9HG9) showing BamA (*grey*), BamB (*orange*), BamD (*brown*), BamC (*green*) and BamE (*purple*). *B*, alphaFold-predicted structure of *B. burgdorferi* BamA (residues 25–821; average predicted local-distance difference test (pLDDT) value of 86.1) superimposed with BamA from *E. coli* (Uniprot: P0A940; *gold*; average pLDDT 92.5; Cα root-mean-square deviation (RMSD) of 3.5 Å). Both proteins are illustrated without the N-terminal signal sequence, and the 16-stranded β-barrel of *B. burgdorferi* BamA is colored grey, while the periplasmic POTRA domains are colored as follows: POTRA1 (residues 28–102), red; POTRA2 (residues 103–180), green; POTRA3 (residues 181–275), blue; POTRA4 (residues 276–353), purple; and POTRA5 (residues 354–430), orange. *C*, superimposed *B. burgdorferi* POTRA domains 1 to 5 (RMSD 1.63–3.05 Å).

High-resolution crystal and cryo-electron microscopy structures have demonstrated that BamA adopts at least two principal conformational states: an inward-open conformation in which the lateral gate between β-strands 1 and 16 is closed and a lateral- or outward-open conformation exposing the barrel interior to the lipid bilayer (8, 9). Cycling between these conformations is essential for function. Mechanistic models propose that OMP assembly is initiated when the C-terminal β-signal of a substrate pairs with the exposed β1 strand of BamA at the lateral gate, followed by sequential β-strand augmentation that generates a transient hybrid BamA-substrate barrel intermediate (10). Resolution of this intermediate and release of the nascent OMP into the outer membrane require coordinated conformational rearrangements within the BamA β-barrel and its periplasmic POTRA domains, coupled to membrane remodelling (8). Importantly, membrane destabilization is not mediated by BamA alone but is a property of the assembled BAM complex (11). Biophysical studies demonstrate that BamA in isolation does not significantly perturb membrane phase behavior, whereas the complete BAM complex lowers the lipid phase transition temperature, indicating cooperative bilayer destabilization by accessory lipoproteins (11). These findings support a model in which accessory proteins contribute not only to substrate recognition and conformational coordination but also to modulation of the lipid environment during OMP insertion.

Conformational transitions within the β-barrel are tightly coupled to movements of the periplasmic POTRA domains. Structural and biophysical analyses indicate that rotation of the POTRA ring correlates with lateral gate opening and closure (10). Moreover, flexibility at the hinge connecting POTRA5 to the β-barrel is required to maintain this coupling, and rigidification of this interface decouples barrel and POTRA motions, impairing BAM activity (12). Together, these findings establish BamA as a dynamic molecular machine in which coordinated domain movements regulate OMP insertion.

In *E. coli*, the BAM complex comprises BamA and four accessory lipoproteins (BamB-E). BamD is the only essential accessory component and plays a central role in coordinating OMP assembly (13, 14) (Fig. 1*A*). BamB interacts primarily with POTRA3 and functions as a scaffolding protein that stabilizes the periplasmic architecture and positions the POTRA domains for productive interaction with substrates and partner proteins (15). BamD independently engages POTRA2 and POTRA5, forming a bipartite bridge that contributes to ring closure and efficient OMP assembly, while BamC and BamE stabilize BamD and modulate overall complex conformation (16, 17).

*B. burgdorferi* encodes a BamA ortholog (BB0795) that is essential for growth, OMP localization, and outer membrane integrity (18). Similar to its *E. coli* counterpart, *B. burgdorferi* BamA (hereafter BbBamA) consists of a 16-stranded transmembrane β-barrel and five POTRA domains (Fig. 1*B*). Although these POTRA domains share a conserved β-α-α-β-β fold, they exhibit low sequence identity (10–22%), suggesting potential functional specialization (Fig. 1*C*).

Subsequent studies identified BB0324 and BB0028 as spirochetal orthologs of BamD and BamB, respectively, and demonstrated that the *B. burgdorferi* BAM complex is more compact than its enterobacterial counterpart, consisting only of BamA, BamB, and BamD (19, 20). Notably, homologs of BamC and BamE are absent, suggesting a distinct periplasmic architecture in spirochetes.

Complicating this architecture further, *B. burgdorferi* encodes a TamB ortholog (BB0794) that interacts with BamA, defining a hybrid BAM-TAM system (21). To place this observation in context, in enterobacteria the translocation and assembly module (TAM) consists of TamA, an outer membrane Omp85-family protein related to BamA, and TamB, a large inner membrane-anchored protein that spans the periplasm to engage TamA (22, 23). In *E. coli*, the TAM is not essential for growth under laboratory conditions but promotes efficient assembly of a subset of outer membrane proteins, particularly autotransporters and other virulence-associated substrates, and contributes to virulence or colonization in multiple pathogens (24). Recent *in vitro* reconstitution experiments further demonstrated that purified *E. coli* TAM can catalyze assembly of several model outer membrane proteins nearly as efficiently as BAM, and that both TamA and TamB are required for activity, supporting the view that TAM can function as an independent β-barrel insertase (25). TamA contains three periplasmic POTRA domains and a 16-stranded β-barrel with a lateral gate analogous to that of BamA, whereas TamB is a 137 kDa periplasmic protein anchored in the inner membrane through an N-terminal signal-anchor helix and is predicted to form an extended β-strand-rich scaffold terminating in a conserved DUF490 domain that interacts with TamA (26). Several studies have shown that residues near the C-terminal region of TamB interact with the N-terminal POTRA domain of TamA, thereby linking the inner and outer membranes through a stable intermembrane complex (27, 28). Structural analysis of the conserved DUF490 region of TamB (residues 963–1138) revealed a taco-shaped β-sheet with a strongly hydrophobic interior, suggesting that TamB may shield hydrophobic β-strands of substrate proteins and act as a periplasm-spanning conduit or chaperone during outer membrane protein biogenesis (29). Consistent with this, mechanistic models propose that TamB delivers partially folded β-strands to TamA, whose lateral gate then promotes membrane insertion through a mechanism related to, but distinct from, BAM (27, 30).

The evolutionary relationship between BAM and TAM is also relevant to the spirochetal system. TamB is more broadly distributed across Gram-negative lineages than TamA, and phylogenetic analyses have suggested that an ancestral BamA-TamB-like partnership may have preceded the emergence of TamA as a specialized Omp85 paralog in Proteobacteria (23, 30). This model is particularly relevant to *B. burgdorferi*, where TamA is absent but TamB interacts with BamA and the DUF490 region binds multiple BamA POTRA domains, especially POTRA2-3, suggesting that interactions between BamA and periplasm-spanning partners contribute to the spatial organization and functional specialization of the spirochetal assembly platform (21).

Across bacterial species, BamA POTRA domains mediate both sequential recognition of nascent OMP substrates and docking of accessory proteins (31). While interactions between BamB and POTRA3 and between BamD and POTRA2/POTRA5 have been experimentally defined in *E. coli* (10, 16, 32), POTRA-domain specificity in *B. burgdorferi*, particularly in light of structural divergence of BamD and absence of BamC and BamE, has not been comprehensively mapped. Furthermore, although crystallization of the N-terminal POTRA region of BbBamA has been reported, high-resolution structures of POTRA1-2 and POTRA2-3 have not been available (33).

Here, we combine AlphaFold3 modeling, mutagenesis, microscale thermophoresis, and crystallography to define the architecture of the spirochetal BAM-TAM complex. Our findings reveal redistribution of accessory contacts, closure of the POTRA ring by BamB and BamD, and structural completion of the BamA β-barrel by TamB. These results establish a reorganized and hybrid assembly platform that expands current understanding of OMP biogenesis in spirochetes and provides a structural framework for future mechanistic and therapeutic studies.

## Results and discussion

### AlphaFold3 predicts reorganized accessory protein docking onto distinct BamA POTRA domains in *B. burgdorferi*

**Figure 2:**
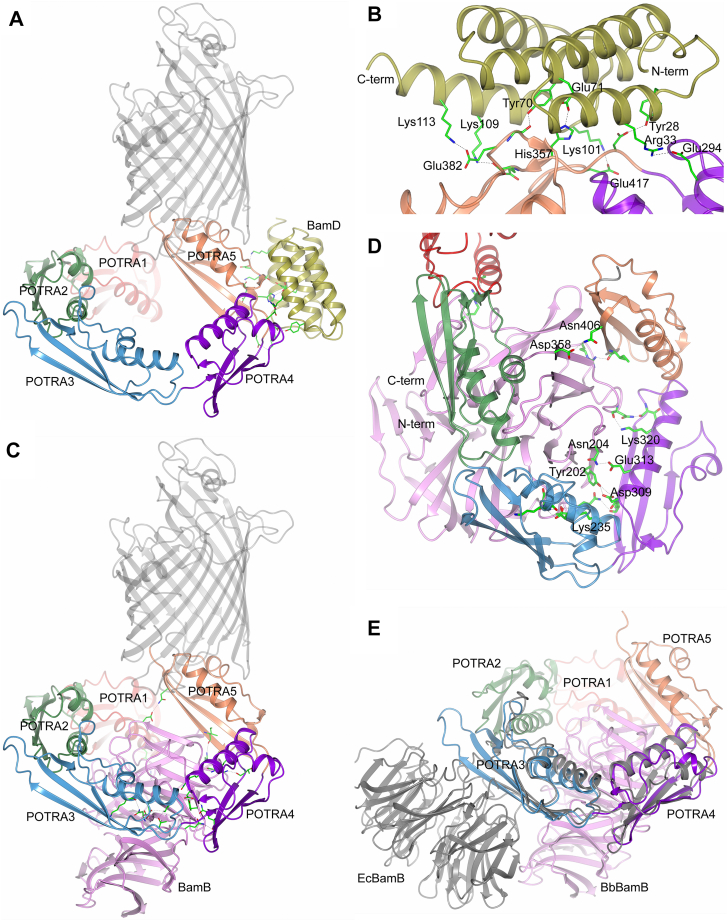
**AlphaFold3 prediction of reorganized BamA accessory protein interactions in*****B. burgdorferi***. *A*, AlphaFold model of *B. burgdorferi* BamD (*gold*) docked to BamA POTRA4-5 (POTRA4, *purple*; POTRA5, *orange*; iptm 0.90 ptm 0.83). POTRA domains are shown in distinct colors for clarity; the BamA β-barrel is shown in transparent *gray*. *B*, Interface close-up of BamD-POTRA4-5 with interacting residues indicated. *C*, alphaFold3 model of *B. burgdorferi* BamB (*pink*) docked to BamA POTRA1-5 (iptm 0.41; ptm 0.48). *D*, interface closeup of BamB contacts with POTRA1, POTRA3, POTRA4, and POTRA5, with interacting side chains indicated. *E*, superposition of the *E. coli* POTRA3 from the BamB-POTRA3 complex (PDB ID: 4XGA; *grey*) with the POTRA3 from the predicted *B. burgdorferi* BamB-POTRA model (BamB *pink*, RMSD 2.79 Å).

To define how the accessory lipoproteins BamD (BB0324) and BamB (BB0028) assemble onto the periplasmic region of BamA (BB0795), multimeric complex predictions were generated using AlphaFold3 (34). Modelling BamA POTRA1-5 with BamD yielded a high-confidence interface (iptm 0.90; ptm 0.83) in which BamD docked primarily onto the membrane-proximal POTRA4-5 domains (Fig. 2*A*). The predicted interface involved extensive contacts between the TPR-containing core of BamD and POTRA4 with additional interactions involving POTRA5 (Fig. 2*B*). In contrast, modelling of BamB with BamA predicted association with POTRA1, POTRA3, POTRA4, and POTRA5 (Fig. 2*C*). This interface was supported by lower confidence metrics (iptm 0.41; ptm 0.48), suggesting either increased conformational flexibility of the POTRA domains or a more transient docking mode (Fig. 2*D*). Notably, the predicted BamB orientation differed from experimentally determined *E. coli* BamA-BamB structures, in which BamB binds primarily to the β-sheet face of POTRA3 near the POTRA2-3 hinge (9, 35, 36) (Fig. 2*E*). Together, these predictions suggest that in *B. burgdorferi*, accessory proteins engage distinct and partially redistributed POTRA surfaces relative to enterobacterial systems. This reorganization likely reflects adaptation to the absence of BamC and BamE and suggests that stabilizing interactions within the periplasmic ring are reassigned across BamB and BamD.

### *B. burgdorferi* BamD represents a truncated ortholog corresponding to the N-terminal Half of *E. coli* BamD

**Figure 3:**
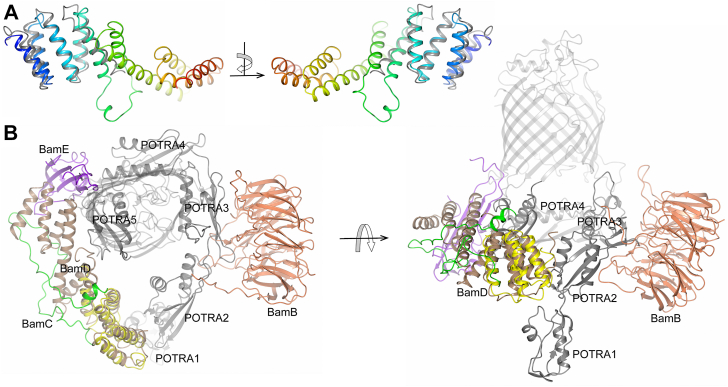
**Structural comparison of*****B. burgdorferi*****and *E. coli* BamD architecture**. *A*, Superposition of BbBamD (residues 23–119; *grey*; average pLDDT 96.3) and EcBamD (Uniprot: P0AC02; residues 30–245; colored from blue at the N-terminus to red at the C-terminus; average pLDDT 94.0), both predicted without signal peptides (RMSD 2.2 Å). (*B*) EcBamD (*brown*) within the *E. coli* BAM complex (PDB ID: 9HG9), including BamA POTRA domains (*grey*), BamC (*green*), and BamE (*purple*), superimposed with BbBamD (*yellow*; RMSD 2.15 Å), illustrated in both top and side views. The overlay highlights that BbBamD corresponds to the N-terminal portion of EcBamD and lacks the C-terminal region involved in interactions with POTRA domains and accessory lipoproteins.

To understand the structural basis of the predicted docking shift, we compared BbBamD with the crystal structure of *E. coli* BamD (hereafter EcBamD). EcBamD comprises ten α-helices arranged into five tetratricopeptide repeat (TPR) motifs that support bipartite engagement with BamA and interactions with accessory lipoproteins (37). Structural superposition revealed that BbBamD (residues 23–119) aligns closely with the N-terminal region of EcBamD (RMSD 2.2 Å), corresponding approximately to the first five α-helices (Fig. 3*A*). BbBamD lacks the C-terminal α-helical region present in EcBamD. As illustrated by superposition of BbBamD onto EcBamD within the *E. coli* BAM complex (Fig. 3*B*), BbBamD corresponds to the N-terminal region of EcBamD and lacks the C-terminal helices required for canonical POTRA2/POTRA5 bridging.

In *E. coli*, BamD establishes two major contacts with BamA: the N-terminal region interacts with POTRA2, while the C-terminal region contacts POTRA5, thereby contributing to closure of the periplasmic ring-like architecture (16) (Fig. 3*B*). This arrangement is further stabilized by BamC and BamE, which interact with BamD and modulate complex conformation (38) (Fig. 3*B*). BamE also contacts POTRA5 and contributes to productive BamA-BamD coordination, and disruption of this coordination impairs OMP assembly (8). This truncation prevents formation of the canonical enterobacterial architecture and correlates with the absence of BamC and BamE homologs in *B. burgdorferi* (Fig. 3*B*). Consistent with this streamlined architecture, AlphaFold3 predicts that BbBamD docks primarily to POTRA4-5 (Fig. 2*A*), repositioning the BamD interaction surface toward the membrane-proximal portion of BamA.

### Divergent BamB docking suggests enhanced plasticity and redistribution of stabilizing contacts

In *E. coli*, BamB is an eight-bladed β-propeller that binds primarily to POTRA3 and contacts the POTRA2-3 hinge (15, 39). BamB contributes to stabilization of the periplasmic assembly platform and increases OMP folding efficiency, but is not essential for viability (8, 17).

In *B. burgdorferi*, AlphaFold3 modeling predicted a broader BamB interaction network involving POTRA1, POTRA3, POTRA4, and POTRA5 (Fig. 2, *C* and *D*). However, the predicted orientation differed from the experimentally observed EcBamB docking geometry, and confidence scores were lower (Fig. 2*E*). Although lower confidence scores suggest conformational heterogeneity, the expanded contact footprint is consistent with a reorganized periplasmic architecture in which stabilizing interactions normally provided by BamC/E are redistributed. Moreover, structural studies have shown that the POTRA array exhibits intrinsic plasticity, particularly at the POTRA2-3 hinge (40), which may contribute to alternative docking arrangements in the spirochetal system. Together, these observations support a model in which POTRA3 remains an interaction hub, but BamB engagement is broadened and potentially more dynamic in *B. burgdorferi*. These observations further support a model in which BamB acts as a flexible scaffold that accommodates structural rearrangements within the POTRA domain array.

### Biochemical validation of the BamD-POTRA4-5 interface

**Figure 4:**
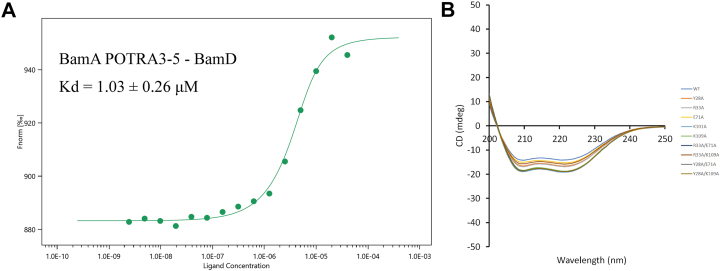
**Biochemical validation of the BamD-POTRA interaction interface**. *A*, MST-binding isotherms for BamD interaction with BamA POTRA3-5. *B*, CD spectra of wild-type BamD and interface mutants.

To experimentally validate the predicted BamD docking site, we expressed and purified soluble BamA fragments encompassing POTRA1-3 and POTRA3-5, along with wild-type BamD and interface-disrupting BamD variants. Microscale thermophoresis (MST) measurements demonstrated that wild-type BamD binds BamA POTRA3-5 with micromolar affinity (Fig. 4*A*). In contrast, no detectable interaction was observed between wild-type BamD and the POTRA1-3 construct under the same MST conditions (Supporting Information). This result is consistent with the AlphaFold3 models, which strongly favored BamD engagement with the membrane-proximal POTRA4-5 region and did not support stable interaction with POTRA1-3. Attempts to express isolated POTRA3-4 and POTRA4-5 fragments resulted predominantly in insoluble protein, suggesting that these domains require adjacent POTRA elements for structural stabilization.

In contrast, BamD mutants targeting residues located at the predicted POTRA4-5 interface did not yield reproducible binding responses comparable to wild-type BamD, supporting the modelled docking surface. The full MST data for all mutant proteins are provided in the Supporting Information. To verify that loss of binding was not caused by global misfolding, circular dichroism (CD) spectroscopy was performed on wild-type BamD and mutant variants. All proteins exhibited similar CD spectra characteristic of predominantly α-helical secondary structure (Fig. 4*B*), consistent with preserved global folding. Together, these data show that BamD binds POTRA3-5 but not POTRA1-3 under the conditions tested, confirming that the AlphaFold-predicted membrane-proximal interface corresponds to the functional BamD docking surface in solution and supports a repositioned BamD coordination mechanism in *B. burgdorferi* relative to the enterobacterial POTRA2/POTRA5 bridging mode.

### Crystal structures of POTRA1-2 and POTRA2-3 define distal architecture and reveal features relevant to accessory protein docking

**Figure 5:**
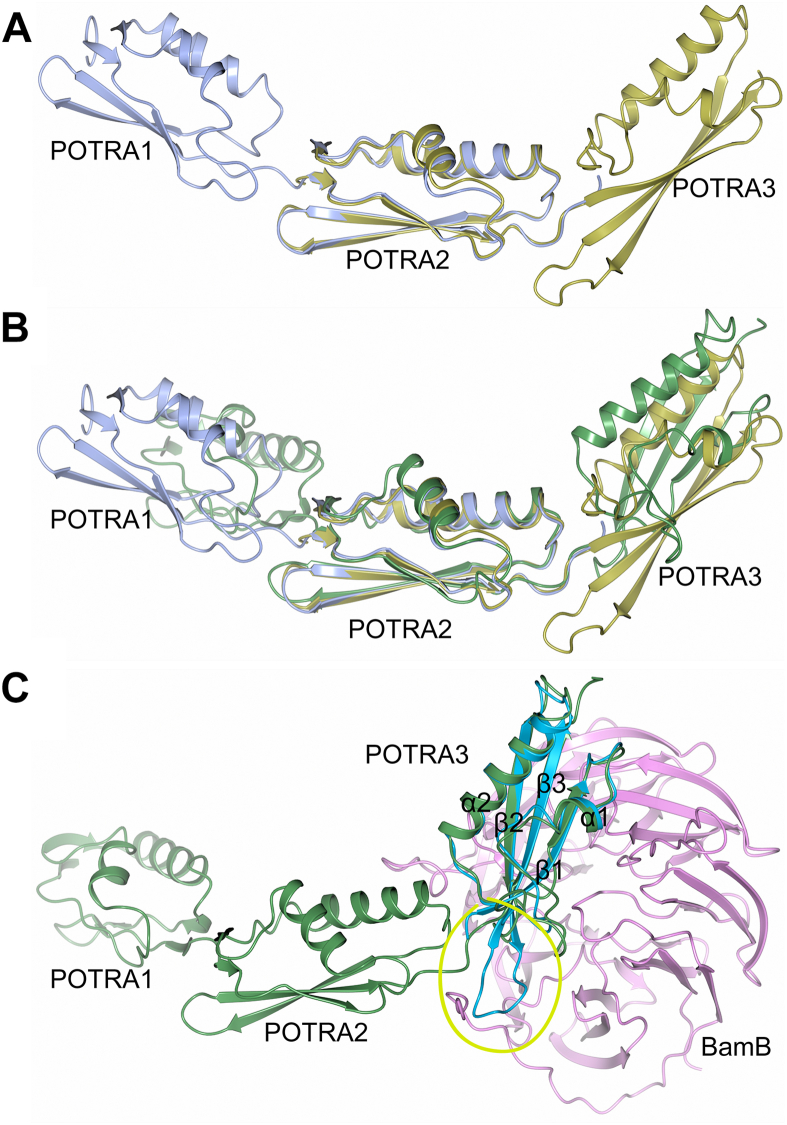
**Crystal structures of BbBamA POTRA domains reveal conformational flexibility and altered BamB docking features**. *A*, Crystal structures of BbBamA POTRA1-2 (*blue*) and POTRA2-3 (*gold*); POTRA2 domains superimposed (RMSD 0.83 Å). *B*, superposition of BbPOTRA1-2 (*blue*), BbPOTRA2-3 (*gold*), and *E. coli* POTRA1-3 (*green*; PDB ID: 9HG9; RMSD 3.62 Å). *C*, superposition of *E. coli* POTRA1-3 in complex with BamB (BamB *pink*) with BbPOTRA3 (*blue*; RMSD 1.32 Å) highlighting an extended BbPOTRA3 loop that interferes with the enterobacterial BamB position.

To provide structural context for accessory protein engagement, we determined crystal structures of BamA POTRA1-2 and POTRA2-3. Both domain pairs adopt the canonical β-α-α-β-β POTRA fold and display distinct interdomain angles (Fig. 5*A*). Superposition with *E. coli* POTRA1-3 indicates that hinge regions between POTRA domains exhibit substantial conformational plasticity. When the POTRA2 domains are superimposed, POTRA1 and POTRA3 adopt shifted orientations (Fig. 5*B*). The surface topology of POTRA3 includes a groove-like β-sheet face compatible with accessory protein docking. However, a detailed comparison of POTRA3 from the *B. burgdorferi* POTRA2-3 crystal structure with POTRA3 from an *E. coli* BamA-BamB complex structure (16) revealed that BbPOTRA3 contains an extended loop between the last two β-strands. This loop sterically interferes with the BamB position observed in *E. coli* (Fig. 5*C*). This structural feature provides a plausible explanation for why AlphaFold3 did not reproduce the canonical enterobacterial BamB docking geometry, even when varying input sequence boundaries, and supports the notion that BamB engagement is reorganized in *B. burgdorferi*. These structural features highlight intrinsic flexibility within the POTRA array and provide a mechanistic basis for the altered accessory protein docking geometry observed in the spirochetal system.

### Structural Context of TamB Supports a Layered Architecture and Reveals β-Barrel Completion with BamA

*B. burgdorferi* encodes a TamB ortholog (BB0794) but lacks TamA. Co-immunoprecipitation previously demonstrated that TamB interacts with BamA, supporting the existence of a hybrid BAM-TAM system (21). Depletion of BB0794 in *B. burgdorferi* causes a striking morphological phenotype in which spirochetes form elongated chains of unseparated cells, accompanied by reduced cell counts during growth; importantly, these defects are rescued by IPTG-induced expression of BB0794 (21). These observations indicate that TamB contributes to normal envelope organization and cell division-linked morphogenesis.

**Figure 6:**
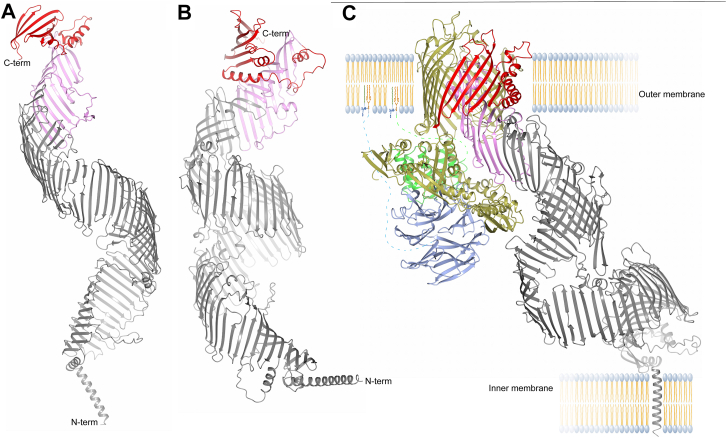
**Structural model of the hybrid BAM-TAM complex in *B. burgdorferi***. *A*, AlphaFold model of full-length EcTamB, with the C-terminal region historically designated DUF490 highlighted (*pink*/*red*)**.***B*, AlphaFold model of full-length BbTamB with corresponding regions highlighted. The structurally distinct terminal segment separated by a loop is shown in *red* (EcTamB residues 1137–1259; BbTamB residues 1285–1453). *C*, updated structural model of the *B. burgdorferi* BAM-TAM complex informed by Hall *et al.* (2024) and the present AlphaFold3 and biochemical analyses showing BamA (*gold*), BamB (*blue*), BamD (*green*), and TamB (*gray*/*pink*/*red*); the TamB C-terminus (*red*) completes the BamA barrel (*gold*).

**Figure 7:**
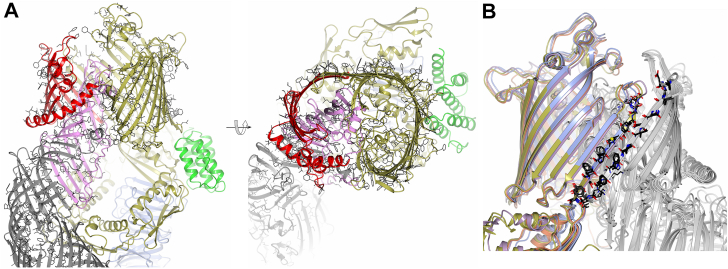
**Conservation of the predicted BamA-TamB hybrid β-barrel architecture across Lyme disease-associated*****Borrelia*****species**. *A,* Side and top views of the predicted BamA-TamB hybrid β-barrel. The C-terminal β-strands of TamB (*red*) align with BamA β1 (*gold*) to complete a continuous β-barrel. Hydrophobic residues lining the membrane-facing exterior surface are highlighted, illustrating compatibility with membrane integration, whereas the interior surface remains hydrophilic. *B*, superposition of AlphaFold-predicted BamA-TamB complexes from *B. burgdorferi* (BamA, *gold*; TamB, *gray*), *B. afzelii* (BamA, *blue*; TamB, *gray*), *B. garinii* (BamA, *orange*; TamB, *gray*), and *B. spielmanii* (BamA, *lilac*; TamB, *gray*). Conserved residues within BamA β1 and the C-terminal β-strand region of TamB are shown as stick representations. The conserved β-strand augmentation architecture is maintained across species, supporting a conserved mode of hybrid barrel formation.

**Figure 8:**
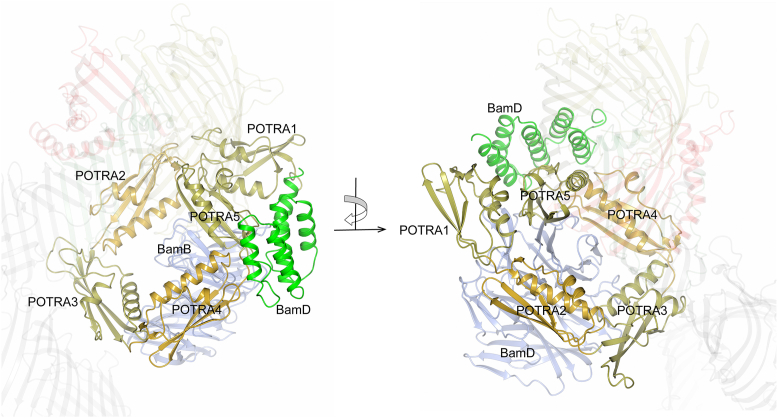
**Proposed model of the reorganized spirochetal BAM-TAM assembly platform**. Structural representation of the BamA periplasmic POTRA domains (*gold*) in the context of the assembled complex. The proximity of POTRA1 and POTRA5 in the predicted architecture suggests that ring closure can be achieved despite the absence of an extended BamD (*green*) bridging interaction. BamD remains associated with the membrane-proximal POTRA4-5 region, while BamB engages multiple POTRA domains, contributing to stabilization of the periplasmic platform. TamB provides additional connectivity through interactions with POTRA2-3 and completes the BamA β-barrel *via* β-strand augmentation, highlighting structural integration of BAM and TAM components.

The DUF490 region of BbTamB (residues 1117–1443) shares low primary-sequence identity with EcTamB but is predicted to adopt a conserved β-strand-rich architecture (41). High-confidence AlphaFold3 prediction of EcTamB reveals approximately 60 β-strands forming an extended, continuous β-sheet scaffold in which one face is predominantly hydrophobic and the exterior surface is largely hydrophilic (Fig. 6*A*). BbTamB is predicted to adopt a highly similar overall architecture despite limited sequence conservation (Fig. 6*B*). While earlier studies described DUF490 as a discrete C-terminal domain (EcTamB residues 923–1259; BbTamB residues 1117–1465), the AlphaFold models instead support a largely continuous fold with a structurally distinct C-terminal segment separated by a loop. This terminal segment comprises an α-helix followed by six β-strands (EcTamB residues 1137–1259; BbTamB residues 1285–1453) (Fig. 6, *A* and *B*). Published data show that the BbTamB DUF490 region interacts with BamA POTRA2-3 (41). Consistent with this, our AlphaFold modelling of the full BAM-TAM complex predicts interactions between TamB and the POTRA platform, particularly involving POTRA3 (and potentially POTRA2). Strikingly, the model further predicts that the six β-strands at the C-terminus of TamB align with BamA β1 to complete a continuous β-barrel with BamA (Fig. 6*C*). The resulting hybrid barrel preserves a hydrophobic exterior compatible with membrane integration and a hydrophilic interior (Fig. 7*A*). To assess conservation of the proposed BamA-TamB interface, orthologs from representative Lyme disease-associated *Borrelia* species (*Borrelia afzelii*, *Borrelia garinii*, and *Borrelia spielmanii*) were analyzed. Sequence alignment revealed that BamA and TamB are highly conserved, with >94% sequence similarity for BamA and >90% for TamB. Notably, residues within the BamA β1 strand and the C-terminal region of TamB are fully conserved, supporting the formation of an equivalent hybrid barrel across these species. Independent AlphaFold modelling of these ortholog pairs consistently reproduced the predicted β-strand augmentation architecture, supporting a conserved interaction mode across spirochetes (Fig. 7*B*). TamB has been proposed to function as a periplasm-spanning scaffold that can shield hydrophobic β-strand surfaces of unfolded substrates within its hydrophobic interior, while exposing hydrophilic surfaces to the aqueous environment (29). In this context, insertion of the TamB C-terminus into the BamA barrel provides a plausible structural mechanism for direct delivery of unfolded β-barrel substrates from the TamB scaffold into BamA without requiring dissociation and re-capture in the periplasm (Fig. 6*C*). While this model provides a structurally attractive mechanism for substrate delivery, it also raises the question of how nascent β-barrel substrates are inserted if the BamA barrel is occupied by the TamB C-terminus. At present, the predicted BamA-TamB β-barrel completion should be considered as a structural model that may represent a transient or regulatory state rather than a permanently occupied insertion pore. Several non-mutually exclusive mechanisms could reconcile TamB engagement with ongoing OMP insertion. First, TamB may undergo transient disengagement or conformational rearrangement during substrate insertion, allowing access to the BamA lateral gate. Second, the TamB C-terminal β-strands may function as a template that facilitates transfer of substrate β-strands directly onto BamA, analogous to a β-strand exchange or handoff mechanism (10, 25). Third, the BamA-TamB interface may form a composite surface that guides substrate entry toward the lateral gate without sterically occluding insertion. These possibilities are consistent with the proposed role of TamB as a dynamic periplasm-spanning scaffold and suggest that β-barrel completion may represent a regulated intermediate in the insertion cycle rather than a static architectural feature. Within this dynamic framework, the integrated model indicates that BamB engages POTRA1, POTRA3, POTRA4, and POTRA5, while BamD docks at POTRA4-5 (Figs. 6*C* and 8), supporting closure of the POTRA ring despite the truncated BbBamD architecture.

Notably, β-barrel completion as an interaction principle has been proposed for the enterobacterial TamA-TamB complex, where TamB completes the TamA barrel (25, 42). Our results extend this concept to a spirochetal BAM-TAM system in which TamB appears to complete the BamA barrel. This provides a mechanistic explanation for conservation of the TamB C-terminus across taxa and suggests that barrel completion may represent a general mode of TAM-mediated engagement with Omp85-family proteins.

Together with the conserved BamD docking and adaptable BamB positioning, these findings support a model in which TamB integration reshapes the global periplasmic architecture without disrupting core interaction interfaces.

### BamD binding is conserved, whereas BamB adapts to global architecture

To assess whether accessory protein interactions are preserved in the context of the full assembly, we compared the BamA-BamB-BamD interfaces observed in pairwise models with those in the BAM-TAM complex (Fig. 6*C*).

The BamD and POTRA4-5 interaction remained unchanged across all models, indicating that BamD establishes a stable and conserved membrane-proximal docking site independent of TamB incorporation. This robustness supports a central role for BamD as a structurally constrained coordination element within the spirochetal BAM complex.

In contrast, BamB displayed modest positional differences in the full complex, which correlate with subtle conformational rearrangements of the BamA POTRA domains. In particular, POTRA1 adopts a slightly shifted orientation toward POTRA5 in the presence of TamB, compared to a more inward-bent conformation in the BamA-BamB-BamD model. Despite these differences, the overall BamB binding region remains preserved.

These observations indicate that BamB exhibits greater positional plasticity and likely functions as a flexible adaptor that accommodates global rearrangements of the periplasmic architecture, whereas BamD defines a stable core interaction site.

### A reorganized and minimal periplasmic coordination platform in *B. burgdorferi*

Collectively, structural modelling, biochemical validation, and crystallographic analysis reveal a reorganized accessory protein docking architecture in the spirochetal BAM complex. In contrast to the enterobacterial system, where BamD bridges POTRA2 and POTRA5 and is stabilized by BamC and BamE, *B. burgdorferi* employs a truncated BamD that docks directly to POTRA4-5 and lacks the C-terminal interaction surface required for BamC/E engagement (Figs. 2*A* and 3*B*). In the spirochetal complex, BamB establishes expanded contacts across POTRA1, POTRA3, POTRA4, and POTRA5, consistent with closure of the POTRA ring and redistribution of stabilizing interactions (Figs. 2*C* and 6*C*). The proximity of POTRA1 and POTRA5 in the predicted architecture may further facilitate ring closure in the absence of a long BamD bridge (Fig. 8). TamB provides additional connectivity *via* POTRA2-3 engagement and completes the BamA barrel through β-strand augmentation, indicating structural integration of BAM and TAM components.

Taken together, these data support a layered and reorganized periplasmic architecture in which BamB and BamD close the POTRA ring, while TamB links the distal POTRA platform to the BamA barrel and potentially provides a direct conduit for substrate delivery (Fig. 6*C*).

The emergence of these noncanonical features may reflect adaptation to the distinctive envelope biology of spirochetes. In *B. burgdorferi*, the outer membrane contains relatively few integral β-barrel proteins but is enriched in surface-exposed lipoproteins, suggesting that the demands on β-barrel assembly differ substantially from those in enterobacteria. A streamlined BAM complex, supplemented by TamB-mediated structural integration, may therefore provide a more efficient or regulated platform for handling a limited set of substrates while maintaining envelope integrity. In addition, spirochetes exhibit elongated cell morphology and undergo coordinated cell division processes that require precise control of envelope biogenesis. The morphological defects observed upon TamB depletion further support a broader role for this protein in maintaining envelope organization. In this context, the hybrid BAM-TAM architecture may facilitate coupling between periplasmic substrate handling, outer membrane insertion, and cellular morphogenesis. Together, these considerations suggest that the noncanonical features described here represent functional adaptations to the unique structural and physiological constraints of spirochetal cell envelopes.

The architectural reorganization observed in *B. burgdorferi* may also have broader implications for outer membrane protein assembly in other spirochetes. Organisms such as *Treponema pallidum* and *Leptospira* spp. possess similarly unusual outer membrane compositions and reduced repertoires of β-barrel outer membrane proteins. Available genomic data suggest that these organisms also encode streamlined BAM components, although the presence and organization of TAM-related factors vary. In this context, the hybrid BAM-TAM architecture described here suggests that spirochetes may employ diverse and noncanonical strategies to compensate for reduced accessory components. However, whether TamB-like proteins engage BamA in a similar β-barrel completion mechanism, and the extent to which BamD architecture is conserved across these species, remain open questions that will require direct structural and functional investigation. These considerations highlight the potential evolutionary plasticity of OMP assembly systems across spirochetes.

Although these conclusions are supported by structural modeling and biochemical validation of BamD interactions, direct structure-guided mutagenesis in native *B. burgdorferi* will be required to fully validate the proposed BamA-TamB interface and its functional role *in vivo*.

## Experimental procedures

### Cloning and protein expression

Genes encoding *B. burgdorferi* BB0795 POTRA domain constructs (POTRA1-5, residues 25–430; POTRA1-2, 25–181; POTRA1-3, 25–273; POTRA2-3, 102–276; POTRA3-4, 181–355; POTRA3-5, 181–430; POTRA4-5, 276–430) were PCR-amplified from *B. burgdorferi* B31 genomic DNA. BB0028 constructs lacking the predicted signal peptide, as determined by SignalP 4.1 (43), were amplified as residues 25 to 349 and 49 to 349 from *B. burgdorferi* B31 genomic DNA. BB0324 (residues 18–119) was amplified from a codon-optimized synthetic gene (BioCat GmbH, Heidelberg, Germany). PCR products were ligated into the pETm-11 expression vector, which encodes an N-terminal 6xHis-tag followed by a TEV protease cleavage site. For protein expression, PCR verified pETm-11 constructs were transformed into *E. coli* BL21 (DE3). Cultures were grown in 2 × TY medium supplemented with kanamycin (50 μg/ml) at 37 °C to mid-log phase. Protein expression was induced with 0.2 mM IPTG, and cultures were incubated for 8 h at 32 °C. Cells were harvested by centrifugation at 5000×*g* for 20 min. Site-directed mutants of BB0324 (Y28A, R33A, E71A, K101A, K109A, R33A/E71A, R33A/K109A, Y28A/E71A, and Y28A/K109A) were generated using the same expression system and purified following the identical protocol as for wild-type BB0324.

### Purification of proteins

Proteins were purified using a protocol similar to that previously described for *B. burgdorferi* CspZ (44). Briefly, *E. coli* cell pellets were resuspended in lysis buffer and disrupted by sonication. Cell debris was removed by centrifugation at 10,000×*g* for 30 min at 4 °C. The clarified lysate was applied to an Ni-NTA agarose gravity-flow column (Qiagen) equilibrated in lysis buffer. Bound 6xHis-tagged proteins were eluted using buffer containing 300 mM imidazole (pH 7.5). To remove the N-terminal 6xHis-tag, eluted proteins were incubated overnight at room temperature with TEV protease. Following cleavage, a second round of Ni-NTA affinity chromatography was performed to remove the cleaved 6xHis-tag and TEV protease. BB0324 and BB0028 were further purified by size-exclusion chromatography on a HiLoad 16/600 Superdex 200 pg column (Cytiva) using an ÄKTA Pure chromatography system (Cytiva). Purified proteins were buffer-exchanged into 15 mM Tris-HCl (pH 8.0) and 50 mM NaCl. Final protein concentrations were as follows: BB0795 POTRA1-5: 4.5 mg/ml; POTRA1-2: 7.5 mg/ml; POTRA1-3: 6.7 mg/ml; POTRA2-3: 5.7 mg/ml; POTRA3-5: 6.2 mg/ml; BB0028 (residues 25–349): 6.2 mg/ml; BB0028 (residues 49–349): 10.5 mg/ml; BB0324: 6.0 mg/ml. Attempts to express and purify BB0795 POTRA3-4 and POTRA4-5 constructs resulted in insoluble protein under the tested conditions.

### MST experiment

Binding interactions between BB0795 POTRA3-5 or POTRA1-3 and BB0324 (wild type and mutant variants) were quantified using microscale thermophoresis (MST) on a Monolith NT.115 instrument (NanoTemper Technologies). BB0795 POTRA3-5 (10 μM) and BB0795 POTRA1-3 were fluorescently labeled separately using the Monolith RED-NHS second Generation labeling kit (NanoTemper Technologies) according to the manufacturer’s instructions. Briefly, proteins were exchanged into labeling buffer (10 mM HEPES pH 7.5, 100 mM NaCl) to remove primary amine-containing components, followed by incubation with the RED-NHS dye at room temperature for 30 min in the dark. Excess dye was removed using the supplied desalting column. Labeled POTRA constructs were diluted to a final concentration of 10 to 20 nM in MST buffer consisting of 10 mM HEPES (pH 7.5), 100 mM NaCl, and 0.05% Tween-20 to prevent adsorption to capillaries. Wild-type BB0324 and all nine mutant variants were prepared in MST buffer and subjected to a 16-point two-fold serial dilution starting from 40 μM. Each dilution was mixed 1:1 (v/v) with labeled POTRA construct to yield a constant fluorescent target concentration and a final ligand concentration range covering the expected binding affinity. Samples were loaded into standard treated capillaries (NanoTemper Technologies). Measurements were performed at 25°C using 20 to 40% LED power. Each binding experiment was performed in triplicate. Thermophoresis data were analyzed using MO. Affinity Analysis software (NanoTemper Technologies). Normalized fluorescence changes were plotted against ligand concentration, and dissociation constants (Kd) were determined by fitting the data to a one-site binding model when binding was observed. For the POTRA1-3 construct, no reproducible concentration-dependent binding response was detected under the tested conditions. For mutant proteins that did not show a reproducible concentration-dependent binding response, no reliable dissociation constant could be determined. Full MST traces for all mutant measurements are provided in the Supporting Information.

### Circular dichroism

Circular dichroism (CD) spectra of wild-type BB0324 and all nine mutant variants were recorded on a Jasco J-1500 spectropolarimeter (Jasco). Measurements were performed as described previously (45) with minor modifications. Protein samples were prepared at a concentration of 20 μM in 15 mM Tris-HCl (pH 8.0) and 20 mM NaCl. Spectra were recorded at 20°C in continuous scanning mode over a wavelength range of 200 to 250 nm using a 2.0 mm pathlength quartz cuvette. Spectra were analyzed using Spectra Manager software (Jasco).

### Site-directed mutagenesis

Site-directed mutagenesis was performed using the pETm-11-BB0324(18–119) construct as a template to generate the following BB0324 variants: Y28A, R33A, E71A, K101A, K109A, R33A/E71A, R33A/K109A, Y28A/E71A, and Y28A/K109A. Mutations were introduced by PCR using mutation-specific complementary primer pairs (Supporting Information) following a standard whole-plasmid amplification protocol. Amplified products were treated with DpnI (New England Biolabs) to digest methylated parental template DNA and enrich for newly synthesized mutant plasmids. DpnI-treated reactions were transformed into *E. coli* XL1-Blue competent cells. Transformants were selected on LB agar plates supplemented with kanamycin (50 μg/ml). Individual colonies were inoculated into LB medium containing kanamycin and grown overnight at 37°C. Plasmid DNA was isolated using a standard miniprep procedure and sequenced to confirm the presence of the desired mutations.

### Crystallization of proteins

Crystallization trials were performed for purified BB0795 POTRA constructs (POTRA1-5, residues 25–430; POTRA1-2, 25–181; POTRA1-3, 25–273; POTRA2-3, 102–276; POTRA3-5, 181–430), BB0028 (residues 25–349 and 49–349), and BB0324 (residues 18–119). Initial crystallization screening was carried out in 96-well sitting-drop vapor diffusion plates using a Tecan Freedom EVO100 liquid-handling workstation (Tecan Group). Drops were prepared by mixing equal volumes (typically 0.4 μl each) of protein solution and reservoir solution. Initial screening was performed using JCSG-plus and Structure Screen 1 and 2 crystallization kits (Molecular Dimensions). Promising conditions were further optimized by varying precipitant concentration and drop ratios. Crystals were obtained for BB0795 POTRA1-2 in 0.2 M sodium malonate dibasic and 20% PEG 3350, and for BB0795 POTRA2-3 in 0.2 M potassium bromide and 17% PEG 3350. Crystals were harvested and cryoprotected by transferring them into reservoir solution supplemented with 10% glycerol prior to flash cooling in liquid nitrogen for storage and subsequent data collection.

### Diffraction data and structure determination

X-ray diffraction data were collected at beamline I03 at the Diamond Light Source. Diffraction images were processed and indexed using XDS and MOSFLM, and integrated intensities were scaled and merged using AIMLESS and SCALA within the CCP4 program suite (46, 47, 48). Initial phases were obtained by molecular replacement using Phaser (49). AlphaFold-predicted models of the respective POTRA domain constructs were used as search templates after removal of low-confidence regions where appropriate. Automated model building was performed using BUCCANEER (50), followed by iterative manual model adjustment in COOT (51). The solvent content in the unit cell was determined by MATTHEWS (52). Crystallographic refinement was performed with REFMAC5 (53). A summary of data collection, refinement, and validation statistics is given in Table 1.

**Table 1:** Statistics for data and structure Quality

Datasetataset	BB0795 (BamA) POTRA1-2	BB0795 (BamA) POTRA2-3
PDB entry	9TRP	9TRQ
Beamline	Diamond beamline I03	Diamond beamline I03
Space group	P 21221	C 2221
Unit cell dimensions		
a (Å)	63.15	38.48
b (Å)	64.59	171.81
c (Å)	89.10	60.67
Wavelength (Å)	0.9762	0.9762
Resolution (Å)	89.10-3.20	85.90-2.30
Highest resolution bin (Å)	3.42-3.20	2.38-2.30
No. of reflections	85,508	126,024
No. of unique reflections	6411	9363
Completeness (%)	100.0 (99.9)	100.0 (100.0)
R_merge_	0.14 (0.37)	0.10 (0.32)
*I/σ* (*I*)	13.8 (7.2)	17.4 (7.4)
Multiplicity	13.3 (14.2)	13.5 (12.7)
Refinement		
R_work_	0.205 (0.324)	0.222 (0.208)
R_free_	0.303 (0.622)	0.276 (0.303)
Average B-factor (Å^2^)		
Overall	51.6	24.6
From Wilson plot	43.6	17.9
No. of atoms		
Protein	2559	1371
Water	0	136
RMS deviations from ideal		
Bond lengths (Å)	0.008	0.015
Bond angles (^o^)	1.567	1.689
Ramachandran outliers (%)		
Residues in most favored regions (%)	84.66	97.04
Residues in allowed regions (%)	15.34	2.96
Outliers (%)	0.00	0.00

Values in parentheses are for the highest resolution bin.

## Ethics statement

This study did not involve experiments with human participants or animals.

## Data availability

The coordinates and structure factors for *B. burgdorferi* BB0795 (BamA) POTRA domains 1 to 2 and BB0795 (BamA) POTRA domains 2 to 3 have been deposited in the Protein Data Bank with the accession numbers 9TRP and 9TRQ, respectively. Sequencing data for BB0324 mutants is included in the supplementary information.

## Supporting information

This article contains supporting information.

## Conflict of interest

The authors declare that they have no conflicts of interest with the contents of this article.
