# Supplementary material for: Structural architecture of a hybrid β-barrel assembly machinery BAM-TAM complex in Borrelia burgdorferi
Source: J Biol Chem. 2026 Jun 19;302(8):113267. doi: 10.1016/j.jbc.2026.113267 (PMC13382580; doi:10.1016/j.jbc.2026.113267)
Supplement: Supporting Figures and Table [file mmc1.docx]

**Structural architecture of a hybrid β-barrel assembly machinery BAM-TAM complex in *Borrelia burgdorferi***

Kalvis Brangulis^a,*^

^a^Latvian Biomedical Research and Study Centre, Ratsupites 1 k-1, LV-1067, Riga, Latvia

* Corresponding author. Mailing address: Latvian Biomedical Research and Study Centre, Ratsupites Street 1 k-1, LV-1067, Riga, Latvia. Phone: +371 20411115

E-mail: kalvis@biomed.lu.lv

**Supporting Information**

**
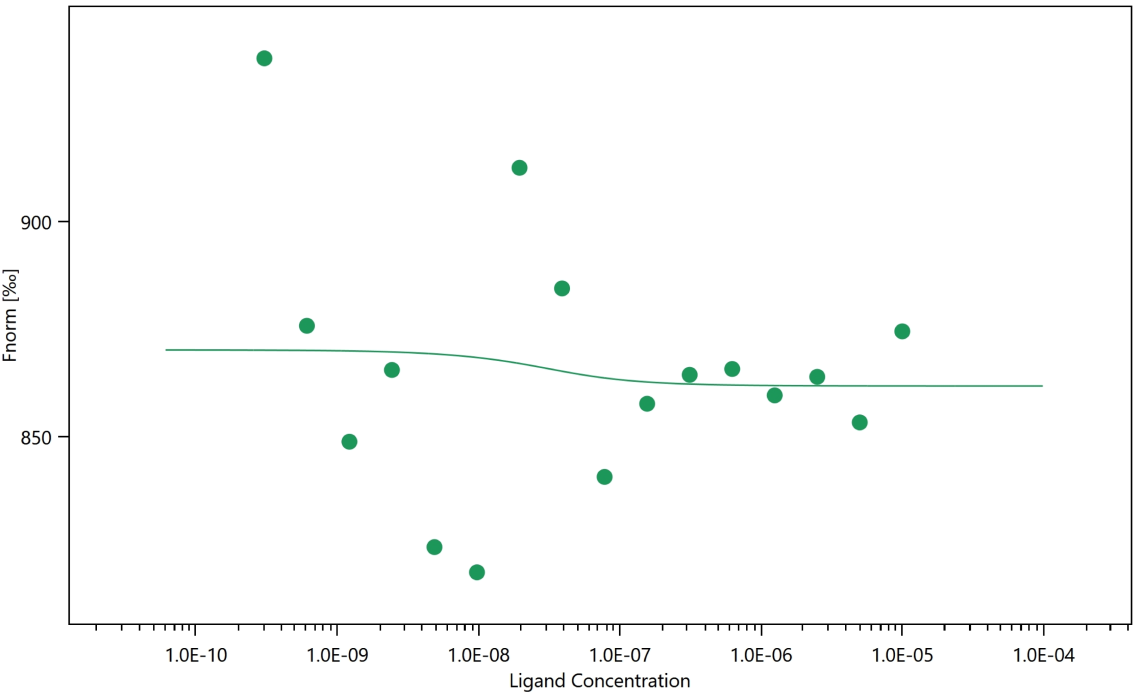
**

**Figure S1.** MST analysis of wild-type BamD with BamA POTRA1-3. (B-J) MST analysis of BamD interface mutants with BamA POTRA3-5. In contrast to wild-type BamD binding to POTRA3-5, POTRA1-3 and the BamD mutant variants did not yield reproducible concentration-dependent MST responses or reliable binding fits under the tested conditions.


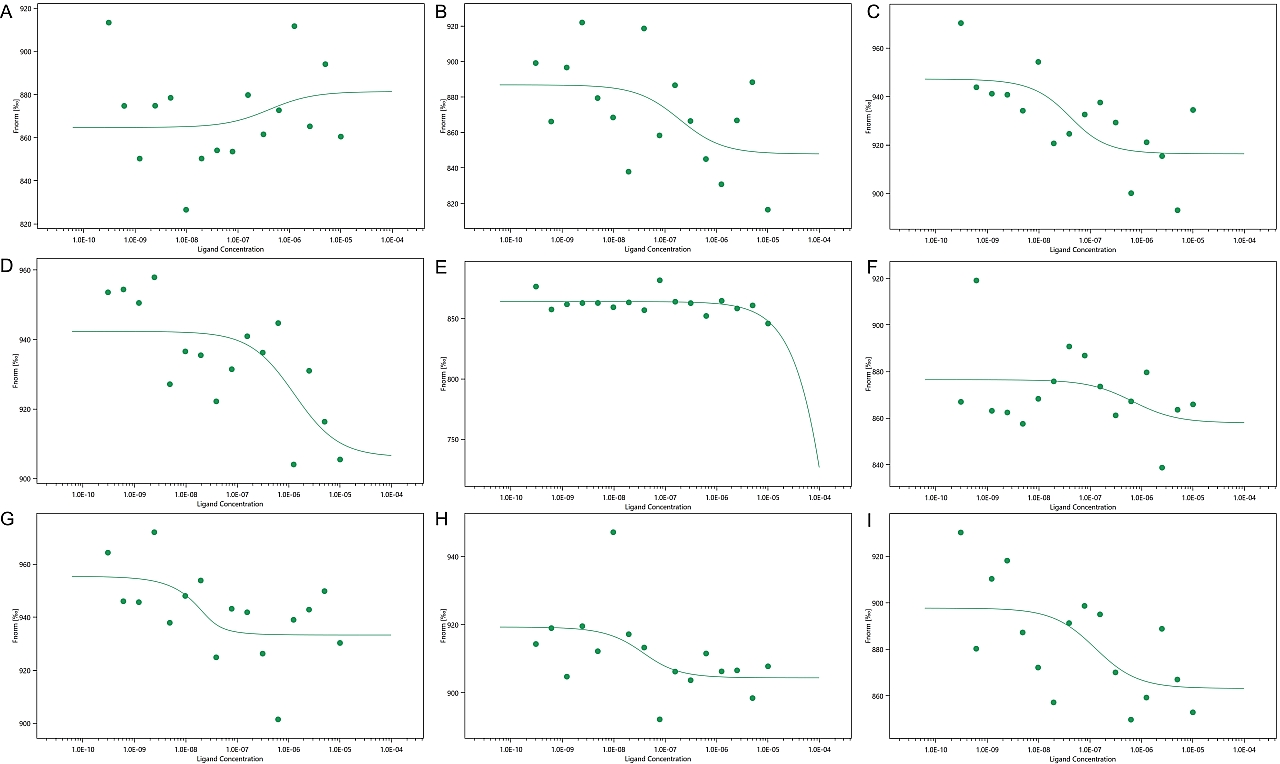


**Figure S2.** (A-I) MST analysis of BamD interface mutants with BamA POTRA3-5. In contrast to wild-type BamD binding to POTRA3-5, the BamD mutant variants did not yield reproducible concentration-dependent MST responses or reliable binding fits under the tested conditions.

**Table S1.** Oligonucleotides used for site-directed mutagenesis.

| Oligonucleotide | Sequence (5’-3’) |
| --- | --- |
| *bamD*  Y28A | AAA GAA ACC CCG GCA GGT GTT TAT CTG CGC GAA GCC CAG AAA and CAG ATA AAC ACC TGC CGG GGT TTC TTT GGT CAG TTT TTC CAG |
| *bamD*  R33A | GGT GTT TAT CTG GCA GAA GCC CAG AAA GCA GTG AAT GTT AAT and TTT CTG GGC TTC TGC CAG ATA AAC ACC ATA CGG GGT TTC TTT |
| *bamD*  E71A | ACC GGT AAA TAT GCA ATT GCC TTT ATC TAT TAC ACC ACC AAT and GAT AAA GGC AAT TGC ATA TTT ACC GGT TGC CAC GAT ATT ACT |
| *bamD*  K101A | ATG GAA ATG CCG GCA TGG ATT AAG CCG CTG GCA AAA AAG ATT and CGG CTT AAT CCA TGC CGG CAT TTC CAT ATT GTT ACC AAT CAG |
| *bamD*  K109A | CCG CTG GCA AAA GCA ATT CTG AAT AAG ATT GAA AAC AAC AAG and CTT ATT CAG AAT TGC TTT TGC CAG CGG CTT AAT CCA TTT CGG |
| *bamD*  R33A/E71A | GGT GTT TAT CTG GCA GAA GCC CAG AAA GCA GTG AAT GTT AAT and TTT CTG GGC TTC TGC CAG ATA AAC ACC ATA CGG GGT TTC TTT; ACC GGT AAA TAT GCA ATT GCC TTT ATC TAT TAC ACC ACC AAT and GAT AAA GGC AAT TGC ATA TTT ACC GGT TGC CAC GAT ATT ACT |
| *bamD*  R33A/K109A | GGT GTT TAT CTG GCA GAA GCC CAG AAA GCA GTG AAT GTT AAT and TTT CTG GGC TTC TGC CAG ATA AAC ACC ATA CGG GGT TTC TTT; CCG CTG GCA AAA GCA ATT CTG AAT AAG ATT GAA AAC AAC AAG and CTT ATT CAG AAT TGC TTT TGC CAG CGG CTT AAT CCA TTT CGG |
| *bamD*  Y28A/E71A | AAA GAA ACC CCG GCA GGT GTT TAT CTG CGC GAA GCC CAG AAA and CAG ATA AAC ACC TGC CGG GGT TTC TTT GGT CAG TTT TTC CAG; ACC GGT AAA TAT GCA ATT GCC TTT ATC TAT TAC ACC ACC AAT and GAT AAA GGC AAT TGC ATA TTT ACC GGT TGC CAC GAT ATT ACT |
| *bamD*  Y28A/K109A | ACC GGT AAA TAT GCA ATT GCC TTT ATC TAT TAC ACC ACC AAT and GAT AAA GGC AAT TGC ATA TTT ACC GGT TGC CAC GAT ATT ACT; CCG CTG GCA AAA GCA ATT CTG AAT AAG ATT GAA AAC AAC AAG and CTT ATT CAG AAT TGC TTT TGC CAG CGG CTT AAT CCA TTT CGG |
